# Rimegepant for vestibular and cochlear symptoms associated with vertigo in a real-world retrospective study

**DOI:** 10.1016/j.isci.2026.117489

**Published:** 2026-09-19

**Authors:** Ziyu Zhai, E. Tian, Wandi Xu, Jun Wang, Zhaoqi Guo, Jingyu Chen, Zhanghong Zhou, Xixi Yu, Ni Zhai, Shun Zhou, Xinbo Gao, Xin Zhang, Yisheng Lu, Yixu Wang, Xin Ma, Sulin Zhang

**Affiliations:** 1 Department of Otolaryngology, Head and Neck Surgery, Tongji Hospital, Tongji Medical College, Huazhong University of Science and Technology, Wuhan 430030, China; 2 Department of Otorhinolaryngology, Head and Neck Surgery, Union Hospital, Tongji Medical College, Huazhong University of Science and Technology, Wuhan 430022, China; 3 Department of Otorhinolaryngology, Head and Neck Surgery, The First Affiliated Hospital, Jiangxi Medical College, Nanchang University, Nanchang 330006, China; 4 Department of Physiology, School of Basic Medicine, Tongji Medical College, Huazhong University of Science and Technology, Wuhan 430030, China; 5 Department of Otorhinolaryngology, Head and Neck Surgery, People’s Hospital, Peking University, Beijing 100871, China

**Keywords:** vertigo, cochlear symptoms, audiovestibular disorders, calcitonin gene-related peptide receptor antagonist, real-world study

## Abstract

Vertigo commonly co-occurs with cochlear symptoms, yet evidence for calcitonin gene-related peptide (CGRP) receptor antagonists across audiovestibular disorders remains limited. We retrospectively evaluated 54 adults with vertigo who received rimegepant 75 mg daily or every other day for 8 weeks. Rimegepant use was associated with reductions in monthly vertigo and headache days, tinnitus severity, and psychological assessment scores, as well as improvements in pure-tone average and aural fullness. By week 8, 94.4% of patients achieved at least a 50% reduction in monthly vertigo days, and the median perceived onset of improvement was 6 days. In exploratory analysis, daily dosing was associated with a greater reduction in monthly vertigo days than alternate-day dosing. These findings provide preliminary real-world evidence supporting prospective controlled evaluation of CGRP-targeted therapy for vertigo-associated vestibular and cochlear symptoms.

## Highlights

• Rimegepant use was associated with fewer monthly vertigo days over 8 weeks
• Tinnitus, aural fullness, and psychological scores decreased during follow-up
• Paired audiometry showed lower hearing thresholds in a 17-patient subset
• Daily dosing was associated with greater vertigo-day reduction than alternate-day dosing

## Graphical abstract

**Figure:**
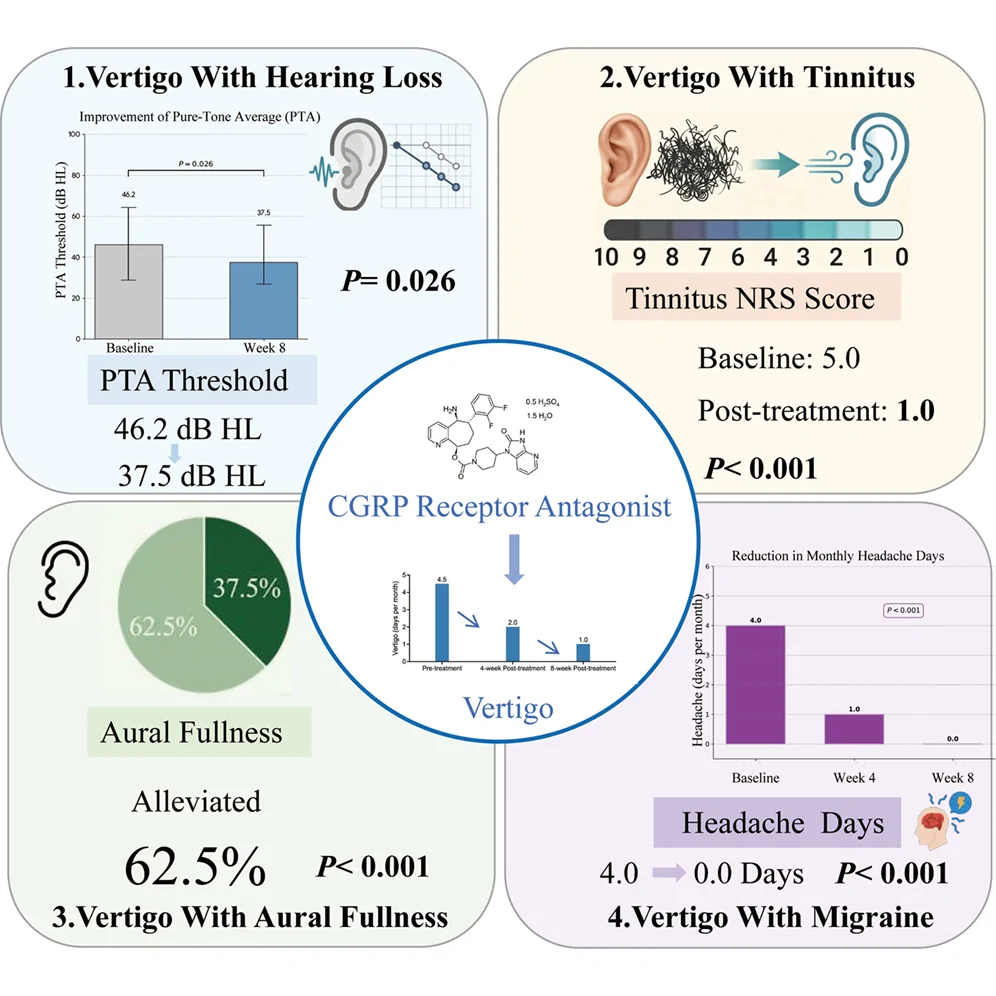

## Teaser

Health sciences; Medicine; Medical specialty; Internal medicine; Neurology

## Introduction

Vertigo is a common and diagnostically challenging symptom in outpatient settings, often exhibiting recurrent or prolonged courses.^1^ Population-based data show 20%–40% of people experience vertigo in their lifetime,^2^ and nearly 25% of these cases are vestibular in origin.^3^ These symptoms are frequently associated with Ménière's disease (MD), vestibular migraine (VM), and other relevant disorders.^4^ Importantly, vertigo rarely occurs in isolation; it is commonly accompanied by a spectrum of cochlear and neurological symptoms, including hearing loss (HL), tinnitus, and headache.^5^^,^^6^^,^^7^ These conditions pose high risks of accidental injury, disability, and mortality^8^^,^^9^^,^^10^; induce psychological harm such as anxiety and depression; cause irreversible organic damage including permanent HL; correlate with cognitive impairments and dementia; and severely impair quality of life.^11^^,^^12^

Currently, treatment for most vertigo-related disorders remains constrained. Treatment strategies predominantly rely on empirical and symptomatic approaches, such as glucocorticoids, microcirculation enhancers, vestibular suppressants, and medications designed to facilitate vestibular compensation.^13^^,^^14^ These therapeutic measures are frequently limited by a lack of well-defined molecular targets and substantial interindividual variability. Consequently, symptoms often persist or remain inadequately controlled, highlighting the inability of conventional therapies to address the complex underlying pathophysiological processes.^15^^,^^16^

Accumulating evidence indicates that dysregulated neurovascular regulation, neurogenic inflammation,^17^^,^^18^ and abnormalities in central-peripheral sensory pathways^19^ are pathological features across multiple otologic disorders. Among these mechanisms, the interplay between the trigeminal-vascular system and vestibulocochlear function^20^^,^^21^ has garnered increasing interest, providing a novel conceptual basis for understanding the intrinsic connections among diverse otological conditions.^22^

The trigeminal system serves as a primary source of multiple pro-inflammatory neuropeptides, including calcitonin gene-related peptide (CGRP),^23^ substance P,^24^ and pituitary adenylate cyclase-activating polypeptide,^25^ among others. CGRP, in particular, is widely acknowledged as a potent vasodilator and a pivotal mediator of neurogenic inflammation. Notably, CGRP is distributed not only within the trigeminal system but also in central vestibular nuclei,^26^ the cochlear spiral ganglion, and inner-ear vasculature,^27^ where it contributes to vasodilation, modulation of neuronal excitability, and neurogenic inflammatory responses. Preclinical investigations suggest that aberrant CGRP expression or excessive release may disrupt inner-ear hemodynamics, alter neural sensitivity, and impair sensory signal integration.^27^ Moreover, in animal models, pharmacological blockade of CGRP receptors has been shown to improve vestibular function by normalizing abnormal synaptic transmission. These observations collectively indicate that CGRP signaling represents a promising therapeutic target for the management of vestibular disorders,^28^ offering a mechanistic foundation for exploring CGRP-targeted interventions in relevant vestibular disorders.

Given these broad effects of CGRP on trigeminal and inner-ear function, CGRP receptor antagonists, a novel class of oral therapeutics, have emerged as effective agents for both acute and preventive migraine treatment.^29^ Recent exploratory clinical studies suggest that these agents may also alleviate inner-ear-related symptoms in patients with comorbid MD and migraine, further supporting the involvement of CGRP in otologic pathophysiology.^30^^,^^31^ Nevertheless, the therapeutic potential of CGRP-targeted approaches for a broader spectrum of audiovestibular disorders remains largely underexplored, with only limited systematic clinical evidence available.^30^

In this retrospective real-world study, we evaluated clinical outcomes in patients treated with the CGRP receptor antagonist rimegepant at an otology clinic for audiovestibular disorders, including vertigo, headache, hearing thresholds, tinnitus, and aural fullness. The aim of this study was to evaluate the clinical outcomes of rimegepant in patients with audiovestibular disorders and to provide initial real-world evidence regarding its potential therapeutic role.

## Results

### Baseline demographic and clinical features

A total of 54 adult outpatients with vertigo were included in this study. The mean age of the participants was 52.1 ± 15.8 years, with a female predominance (79.6%). At baseline, the median number of monthly vertigo days was 4.5 (interquartile range [IQR], 4.0–6.0). According to concomitant symptoms, patients were categorized into subgroups, including HL (*n* = 17), tinnitus (*n* = 37), aural fullness (*n* = 32), and migraine (*n* = 33). Detailed demographic and clinical characteristics are presented in Table 1.

**Table 1:** Baseline demographic and clinical characteristics of the study population

	Total vertigo (*n* = 54)	Vertigo with HL (*n* = 17)	Vertigo with tinnitus (*n* = 37)	Vertigo with fullness (*n* = 32)	Vertigo with migraine (*n* = 33)
Age	52.1 (15.8)	48.7 (14.9)	52.6 (14.5)	53.4 (13.9)	51.1 (18.2)
Sex, *n* (%)					
Male	11 (20.4%)	7 (41.2%)	10 (27.0%)	7 (21.9%)	3 (9.1%)
Female	43 (79.6%)	10 (58.8%)	27 (73.0%)	25 (78.1%)	30 (90.9%)
BMI, kg/m^2^	24.0 (3.3)	24.2 (4.0)	24.3 (3.6)	24.4 (3.7)	24.3 (3.2)
Duration, years	1.0 [0.2–5.2]	0.3 [0.2–2.0]	2.0 [0.3–5.5]	2.0 [0.3–6.0]	2.0 [0.3–5.0]
Vertigo (days per month)	4.5 [4.0–6.0]	3.6 (1.9)	4.0 [3.0–6.0]	4.5 [4.0–6.0]	5.0 [4.0–6.0]
Headache (days per month)	4.0 [4.0–5.0]^a^	3.3 (1.8)^a^	4.0 [3.0–5.0]^a^	4.0 [3.0–5.0]^a^	4.0 [4.0–5.0]
Aural fullness, *n* (%)	32 (59.3%)	12 (70.6%)	31 (83.8%)	32 (100.0%)	19 (57.6%)
Motion sickness, *n* (%)	12 (22.2%)	3 (17.6%)	10 (27.0%)	10 (31.3%)	10 (30.3%)
Family history of vertigo, *n* (%)	11 (20.4%)	1 (5.9%)	8 (21.6%)	8 (25.0%)	9 (27.3%)
Family history of migraine, *n* (%)	7 (13.0%)	0 (0.0%)	6 (16.2%)	6 (18.8%)	6 (18.2%)
Tinnitus NRS	5.0 [3.3–6.0]^a^	5.0 [3.0–6.0]^a^	5.0 [4.0–6.0]	5.0 [3.5–6.0]^a^	5.0 [3.0–6.0]^a^
SAS	32.5 [31.6–37.5]	33.2 (4.1)	32.5 [32.5–35.0]	32.5 [30.9–35.0]	32.5 [28.8–38.8]
SDS	30.6 [27.5–37.5]	32.5 (4.8)	30.0 [27.5–37.5]	29.4 [27.5–37.8]	31.2 [27.5–38.8]
SCL-90	130.0 [128.0–134.8]	131.0 [128.0–134.0]	131.0 [128.0–135.0]	131.0 [128–136]	129.0 [122–134]

Data are presented as *n* (%), mean (SD), or median [IQR]. SD, standard deviation; HL, hearing loss; BMI, body mass index; NRS, numerical rating scale; SAS, self-rating anxiety scale; SDS, self-rating depression scale; SCL-90, Symptom Checklist-90.

a Headache days and tinnitus NRS: median [IQR]/mean (SD) for participants with corresponding symptoms in each group.

### Changes in vestibular symptoms and psychological status following rimegepant treatment

During the 8-week follow-up period, monthly vertigo days and psychological assessment scores were lower than at baseline. The median number of monthly vertigo days decreased significantly from 4.5 (IQR, 4.0–6.0) at baseline to 2.0 (IQR, 1.0–3.0) at week 4 and further to 1.0 (IQR, 0.0–2.0) at week 8 (*p* < 0.001, Table 2). At week 4, 79.6% (43/54) of patients achieved a ≥50% reduction in monthly vertigo days, increasing to 94.4% (51/54) at week 8. Psychological assessment scores, including the self-rating anxiety scale (SAS), self-rating depression scale (SDS), and Symptom Checklist-90 (SCL-90), also decreased significantly compared with baseline (all *p* < 0.05, Table 2).

**Table 2:** Changes in primary and secondary outcomes after rimegepant treatment

Group	Outcomes	Baseline	Week 4	Effect vs. baseline (95% CI)	*p* value	Week 8	Effect vs. baseline (95% CI)	*p* value
Total vertigo	primary outcomes							
	vertigo (days per month)	4.5 [4.0–6.0]	2.0 [1.0–3.0]	−3.0 (−3.5 to −2.5)	<0.001	1.0 [0.0–2.0]	−4.0 (−5.0 to −3.5)	<0.001
	secondary outcomes							
	SAS	32.5 [31.6–37.5]	–	–	–	32.5 [27.5–37.5]	−2.5 (−5.0 to 0.0)	0.031
	SDS	30.6 [27.5–37.5]	–	–	–	30.0 [26.3–36.3]	−1.9 (−3.1 to 0.0)	0.032
	SCL-90	130.0 [128.0–134.8]	–	–	–	129.5 [122.0–134.0]	−6.0 (−10.0 to −1.0)	0.031
Vertigo with	primary outcomes							
HL	PTA (dB HL)	46.2 [28.8–64.4]	–	–	–	37.5 [26.9–55.6]^a^	−4.4 (−8.1 to 0.0)	0.026
Tinnitus	tinnitus NRS	5.0 [4.0–6.0]	2.0 [1.0–3.0]	−2.5 (−3.0 to −2.0)	<0.001	1.0 [0.0–3.0]	−3.0 (−3.5 to −2.5)	<0.001
Aural fullness	aural fullness	32 (100.0%)	19 (59.4%)	–	<0.001	12 (37.5%)	–	<0.001
Migraine	headache (days per month)	4.0 [4.0–5.0]	1.0 [1.0–2.0]	−3.0 (−3.5 to −2.5)	<0.001	0.0 [0.0–1.0]	−4.0 (−4.5 to −3.5)	<0.001

Data are presented as *n* (%), mean (SD), or median [IQR]. Changes from baseline are reported as Hodges-Lehmann estimates of paired differences (follow-up minus baseline) with 95% CIs. Hodges-Lehmann estimates are expressed in the original units of the corresponding outcomes. HL, hearing loss; PTA, pure-tone average.

a Post-treatment audiometry was conducted during the follow-up period; NRS, numerical rating scale; SAS, self-rating anxiety scale; SDS, self-rating depression scale; SCL-90, Symptom Checklist-90; –, not applicable.

### Changes in cochlear symptoms and migraine following rimegepant treatment

Among patients included in the hearing analysis, the pure-tone average (PTA) was lower at follow-up than those at baseline, decreasing from 46.2 (IQR, 28.8–64.4) dB HL to 37.5 (IQR, 26.9–55.6) dB HL (Table 2; *p* = 0.026; Figure 1). Tinnitus severity also improved during follow-up, with the median numerical rating scale (NRS) score decreasing to 1.0 (IQR, 0.0–3.0) at week 8 (*p* < 0.001). The proportion of patients reporting aural fullness decreased from 100% (32/32) at baseline to 37.5% (12/32) at week 8 (*p* < 0.001). Monthly migraine days also declined, with the median decreasing from 4.0 (IQR, 4.0–5.0) to 0.0 (IQR, 0.0–1.0) at week 8 (*p* < 0.001).

**Figure 1:**
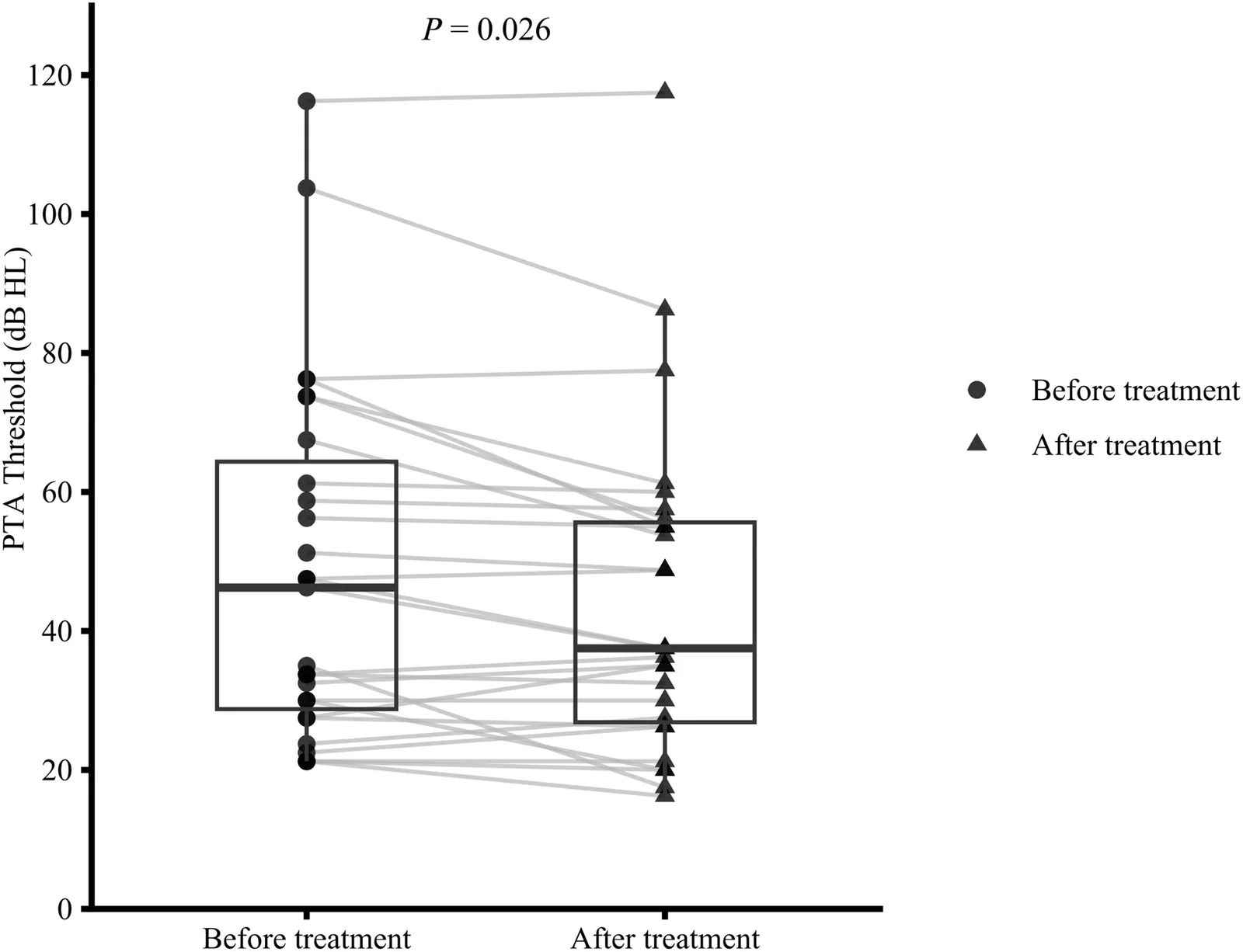
Changes in PTA thresholds before and after rimegepant treatment Each point represents one patient with paired audiometric data (*n* = 17). The *p* value was calculated using a two-sided paired Wilcoxon signed-rank test. PTA, pure-tone average.

### Exploratory patient-reported global improvement

As recorded during the week 2 follow-up, patients reported a median time to perceived symptom improvement of 6.0 days (IQR, 4.5–8.0). At week 8, 81.5% of patients reported either “marked” or “moderate” overall improvement according to the exploratory patient-reported global assessment (Table 3). At baseline, the once-daily (qd) or every-other-day (eod) groups were comparable, with no significant differences in demographic or clinical characteristics (all *p* > 0.05, Table 4). Compared with the eod regimen, the qd regimen was associated with a greater reduction in monthly vertigo days (*β* = −1.896, 95% confidence interval [CI]: −3.67 to −0.12, *p* = 0.042, Figure 2). Dosing-regimen associations for aural fullness are shown in Figure 3. During the 8-week follow-up, no clinically apparent treatment-related adverse events were documented in the available medical records.

**Table 3:** Patient-reported overall symptom improvement after treatment

Time point	Marked improvement (*n*, %)	Moderate improvement (*n*, %)	Mild improvement (*n*, %)	No improvement (*n*, %)
Week 4	29 (53.7%)	13 (24.1%)	12 (22.2%)	0 (0.0%)
Week 8	28 (51.9%)	16 (29.6%)	10 (18.5%)	0 (0.0%)

**Table 4:** Baseline demographic and clinical characteristics of patients in the qd and eod medication groups

	qd (*n* = 28)	eod (*n* = 26)	*p* value
Age	51.4 ± 14.2	52.8 ± 17.6	0.748
Sex, *n* (%)	–	–	1.000
Male	6 (21.4%)	5 (19.2%)	–
Female	22 (78.6%)	21 (80.8%)	–
BMI, kg/m^2^	23.1 ± 3.0	25.0 ± 3.5	0.052
Duration, years	1 (0.3, 5.0)	2.0 (0.1, 6.0)	0.212
Vertigo (days per month)	4.5 (4.0, 6.5)	4.7 ± 2.4	0.072
Headache (days per month)	4.0 (3.5, 5.0)	4.3 ± 1.4	0.566
Aural fullness, *n* (%)	15 (53.6%)	17 (65.4%)	0.545
Motion sickness, *n* (%)	5 (17.9%)	7 (26.9%)	0.636
Family history of vertigo, *n* (%)	6 (21.4%)	5 (19.2%)	1.000
Family history of migraine, *n* (%)	4 (14.3%)	3 (11.5%)	1.000
Tinnitus NRS	4.9 ± 1.9	4.6 ± 1.6	0.600
SAS	32.5 (28.4, 35.3)	35.0 (32.5, 39.7)	0.053
SDS	28.8 (26.2, 33.8)	32.5 (28.8, 38.4)	0.070
SCL-90	128.0 (121.8, 134.0)	131.5 (128.0, 139.0)	0.102

Data are presented as *n* (%), mean (SD). BMI, body mass index; NRS, numerical rating scale; SAS, self-rating anxiety scale; SDS, self-rating depression scale; SCL-90, Symptom Checklist-90.

**Figure 2:**
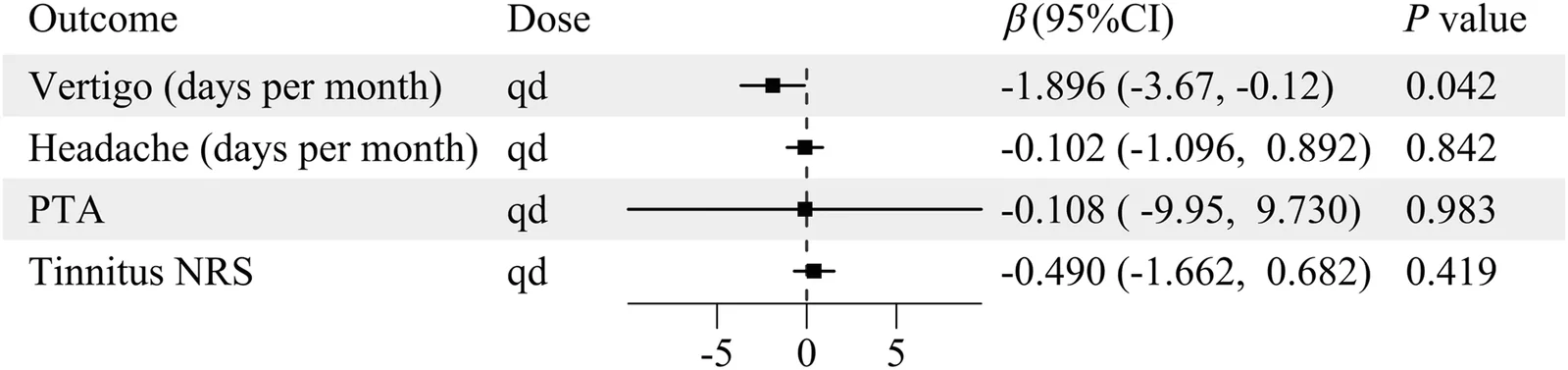
Response of primary outcomes to rimegepant dosing regimens Forest plot showing adjusted *β* coefficients with 95% CIs for vertigo (days per month, *n* = 54), headache (days per month, *n* = 33), PTA (*n* = 17), and tinnitus NRS score (*n* = 37) for qd versus eod dosing. PTA, pure-tone average; NRS, numerical rating scale; qd, once daily; eod, every other day; CI, confidence interval.

**Figure 3:**
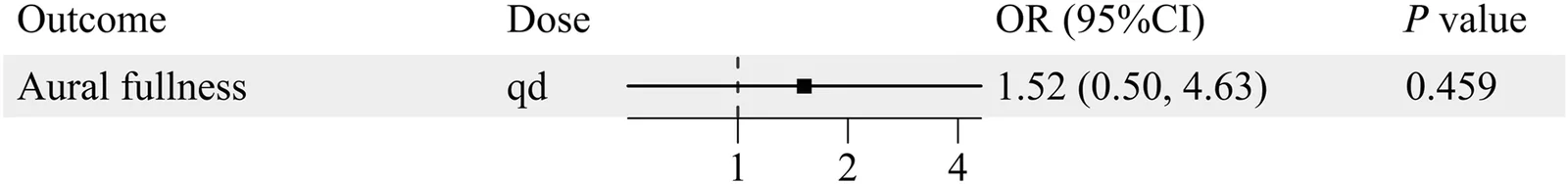
Response of aural fullness to rimegepant dosing regimens The dosing regimen association with aural fullness improvement was estimated using adjusted logistic regression (*n* = 32 patients). qd, once daily.

## Discussion

In this exploratory real-world retrospective study, treatment with rimegepant was associated with improvements across multiple audiovestibular outcomes in patients presenting with vertigo, including reductions in monthly vertigo days, improvements in cochlear symptoms (HL, tinnitus, and aural fullness), reductions in monthly migraine days, and improvements in psychological status. These findings provide preliminary real-world evidence supporting further investigation of CGRP receptor antagonists in patients with audiovestibular symptoms. To our knowledge, this is among the first clinical studies to comprehensively evaluate the effects of a CGRP receptor antagonist on combined vestibular and cochlear manifestations in patients presenting with vertigo. In addition, our exploratory dose-response analysis suggests that dosing frequency may be associated with differences in clinical outcomes, providing preliminary evidence to guide the design of future prospective studies.

### Potential role of CGRP receptor antagonists in vestibular symptoms

The present study found that rimegepant use was associated with reductions in monthly vertigo days over the 8-week follow-up period. These findings provide preliminary real-world evidence supporting further investigation of CGRP receptor antagonists for vestibular symptoms. Our findings are consistent with previous reports. A prospective study reported that rimegepant reduced monthly vestibular symptom days by 8.48 within one month,^32^ while a placebo-controlled trial of galcanezumab (another CGRP-targeted agent) showed significant reductions in dizzy days among patients with VM^33^; a prospective observational cohort study further demonstrated that anti-CGRP monoclonal antibodies yielded a high vertigo response rate and reduced mean monthly vestibular symptom days from a baseline of 10.3 ± 1.9 to 0.8 ± 0.3 in patients with VM.^34^ Additionally, clinical observations suggest CGRP receptor antagonists may alleviate symptoms of Menière’s disease, supporting a link between CGRP overexpression and otologic disorder pathogenesis.^30^^,^^35^ These observations are biologically plausible and are supported by current understanding of CGRP signaling in VM. As a key trigeminal neuropeptide expressed in vestibular and inner-ear tissues, CGRP regulates vasodilation, neuronal excitability, and inflammatory responses, and its abnormal release is thought to disrupt inner-ear blood flow and neural sensitivity, thereby potentially contributing to vestibular symptoms.^29^^,^^35^

### Potential role of CGRP receptor antagonists in cochlear symptoms

To further explore the potential role of CGRP receptor antagonists in cochlear symptoms, this study extended its focus beyond vestibular symptoms to comprehensively evaluate cochlear-related outcomes via combined audiovestibular manifestations, thereby enriching the understanding of CGRP-targeted therapy in otological disorders.^36^ Although the hearing analysis was limited to patients with paired pre- and post-treatment audiometric data, treatment was associated with improvements in hearing thresholds, together with improvements in tinnitus and aural fullness. These findings on hearing improvement are corroborated by relevant literature. A prospective study reported that CGRP receptor antagonists could improve patients’ auditory function,^32^ with statistically significant improvement in the air-conduction threshold of the right ear, which is consistent with the hearing-threshold change observed in our study. The consistency between our findings and previous reports supports further investigation of the potential role of CGRP-targeted therapy in cochlear symptoms.^32^^,^^35^

### Rapid clinical improvement following CGRP receptor antagonist treatment

Patients reported a median time to perceived overall symptom improvement of 6 days, and 79.6% of patients achieved a ≥50% reduction in monthly vertigo days by week 4, increasing to 94.4% by week 8. Unlike CGRP monoclonal antibodies, which reach peak plasma concentration around 5 days and have a prolonged half-life of approximately 27 days favoring preventive effects,^37^ rimegepant, as a small-molecule CGRP receptor antagonist, achieves effective plasma levels rapidly, allowing earlier pharmacological activity. Compared with traditional acute migraine therapies, including triptans, nonsteroidal anti-inflammatory drugs, and ditans, rimegepant demonstrates a comparable time to peak concentration (approximately 1.0–1.8 h)^38^ but a longer elimination half-life (approximately 11 h versus 2.0–5.7 h),^39^^,^^40^^,^^41^ supporting more sustained exposure. While conventional agents primarily provide short-term symptom relief via nonspecific anti-inflammatory effects or 5-HT_1_F receptor agonism,^41^^,^^42^ rimegepant’s direct CGRP receptor antagonism combined with its prolonged effective concentration may contribute to the relatively rapid onset and sustained symptom improvement observed in this study.

### Association between dosing CGRP receptor antagonists and outcomes

The exploratory rimegepant comparison indicated that qd dosing was associated with a greater reduction in monthly vertigo days than eod dosing. One possible explanation is that daily administration may maintain more stable circulating drug concentrations, potentially contributing to sustained pharmacological activity. Although these findings should be interpreted with caution given the exploratory nature of the analysis, they suggest that dosing frequency may represent an important determinant of clinical response and, therefore, warrants further investigation. These observations provide preliminary evidence for optimizing dosing strategies in future prospective studies of CGRP receptor antagonists for audiovestibular disorders.

### Potential role of CGRP receptor antagonists in psychological outcomes

In addition to evaluating vestibular and cochlear outcomes, the present study incorporated standardized psychological assessments, providing a more comprehensive evaluation of patient-reported outcomes. Improvements were observed in SAS, SDS, and SCL-90 scores following treatment. These findings are generally consistent with previous studies investigating CGRP-targeted therapies. Notably, a real-world retrospective study demonstrated that galcanezumab significantly reduced both depression and anxiety scores, independent of changes in migraine frequency, suggesting a potential direct psychological effect of CGRP-targeted agents.^43^ Additionally, a large prospective registry study of erenumab found that treatment responders (≥50% reduction in monthly migraine days) showed significant improvements in anxiety and depression, with benefits seen regardless of baseline psychiatric history.^44^

Based on these findings, the improvements in psychological outcomes observed in our study may reflect multiple contributing factors, including reduced symptom burden following improvements in audiovestibular symptoms and the potential influence of CGRP pathway modulation. This improvement is consistent with the core logic of our team’s unreported six-dimensional classification model for vertigo diagnosis and treatment (encompassing autoimmunity, migraine, otogenic, cognition, anxiety/depression, and sleep), which emphasizes the integrated regulation of emotional dimensions in vertigo management.

### Limitations of the study

This study has several limitations. Its retrospective, single-center design and the absence of a placebo-controlled comparison group limit causal inference. Safety assessments relied on routine clinical chart documentation without standardized prospective adverse event recording or serial hepatic function monitoring; mild unrecorded adverse effects cannot be excluded due to retrospective design limitations. In addition, audiometric analyses were restricted to a small subset of 17 patients with complete paired pre- and post-treatment audiometry; all hearing-related findings should be interpreted with extreme caution due to limited sample size and non-standardized follow-up timing. The hearing analysis was based on this small subgroup of patients with paired audiometric data, and diagnosis-specific subgroup and dose-response analyses should be regarded as exploratory.

Future prospective, multicenter studies should incorporate placebo-controlled designs, standardized objective vestibular assessments, serial audiometric evaluation, and validated disease-specific outcome measures (e.g., vestibular migraine patient assessment tool and handicap inventory [VM-PATHI]), together with systematic safety monitoring and adequately powered diagnosis-specific analyses, to further evaluate the efficacy, safety, and optimal dosing strategies of CGRP receptor antagonists in audiovestibular disorders.

In this exploratory real-world retrospective study, treatment with rimegepant was associated with improvements across multiple audiovestibular outcomes, including vertigo, HL, tinnitus, aural fullness, migraine, and psychological status. Clinical improvement was observed within the first week after treatment initiation, while the exploratory comparison of rimegepant dosing suggested that dosing frequency may influence clinical outcomes. As one of the first real-world studies to comprehensively evaluate the association of a CGRP receptor antagonist with both vestibular and cochlear manifestations in patients presenting with vertigo, this study extends the current clinical evidence beyond VM and provides preliminary real-world evidence supporting further prospective evaluation of CGRP-targeted therapy in audiovestibular disorders.

## Resource availability

### Lead contact

Further information and requests for resources and reagents should be directed to and will be fulfilled by the lead contact, Sulin Zhang (sulin_zhang@hust.edu.cn).

### Materials availability

This study did not generate new unique reagents.

### Data and code availability

• Data reported in this paper will be shared by the lead contact upon request.
• This paper does not report original code.
• Any additional information required to reanalyze the data reported in this paper is available from the lead contact upon request.

## Acknowledgments

This work was supported by grants from the 10.13039/501100012166National Key Research and Development Program of China (2023YFC2508403, 2023YFC2508401, 2023YFC2508002, and 2025YFF1501300), the 10.13039/501100001809National Natural Science Foundation of China (82371168 and 82171152), and the Beijing Natural Science Foundation (7252145).

## Author contributions

The study was designed by S. Zhang, Y.L., Z. Zhai, and E.T. Clinical data collection, statistical analysis, and manuscript drafting were performed by Z. Zhai and E.T. Y.L., Y.W., S. Zhang, and X.M. provided supervision and final check. All authors discussed the results and participated in manuscript preparation and editing.

## Declaration of interests

The authors declare no conflicts of interest.

## STAR★Methods

### Key resources table

**Table:** 

REAGENT or RESOURCE	SOURCE	IDENTIFIER
**Chemicals, peptides, and recombinant proteins**		

Rimegepant orally disintegrating tablets, 75 mg	Pfizer Investment Co., Ltd.	NMPA Approval No. HJ20240004

**Software and algorithms**		

R statistical software	R Foundation for Statistical Computing	Version 4.4.0; https://www.r-project.org/

### Experimental model and study participant details

This retrospective study included 54 adult outpatients treated with rimegepant at the Otology Clinic of Union Hospital, Tongji Medical College, Huazhong University of Science and Technology, between February and December 2025. Patients were categorized according to the presence of HL, tinnitus, aural fullness, and migraine for the corresponding subgroup analyses. The study was approved by the Medical Ethics Committee of Union Hospital, Tongji Medical College, Huazhong University of Science and Technology (No. 2024–0295-01), and informed consent was obtained from all participants.

The cohort comprised 11 male and 43 female participants (mean age, 52.1 ± 15.8 years). Participants were not randomized to treatment groups; treating physicians selected once-daily (*n* = 28) or every-other-day (*n* = 26) dosing in routine care. Symptom-defined subgroups included HL (*n* = 17), tinnitus (*n* = 37), aural fullness (*n* = 32), and migraine (*n* = 33).

Before inclusion in this retrospective analysis, all patients underwent routine clinical evaluation as part of standard medical care. Diagnostic assessment included a detailed medical history, physical examination, positional testing when clinically indicated, vestibular function testing, including videonystagmography (VNG), video head impulse testing (vHIT), and other neurotological assessments as clinically indicated. Audiological evaluation included pure-tone audiometry (air- and bone-conduction thresholds) and tympanometry. Final diagnoses were established by experienced otologists according to internationally recognized diagnostic criteria and were confirmed or refined during routine clinical follow-up when appropriate.

Patients were diagnosed according to the following established guidelines: (1) VM or probable VM (pVM) according to the 2022 Bárány Society criteria^45^; (2) MD or probable MD (pMD) according to the 2020 American Academy of Otolaryngology-Head and Neck Surgery criteria^46^; and (3) HL according to the 2021 World Health Organization hearing loss grading system.^47^ For the hearing analysis, only ears with sensorineural hearing loss and a Type A tympanogram were included, whereas ears with conductive or mixed hearing loss were excluded.

Inclusion criteria were as follows: (1) age ≥18 years; (2) fulfillment of at least one of the aforementioned diagnostic criteria; (3) treatment with rimegepant as part of routine clinical management; and (4) availability of regular follow-up data for assessment of treatment outcomes.

Exclusion criteria were as follows: (1) severe cardiovascular disease; (2) pregnancy or lactation; (3) documented psychiatric disorders that could interfere with clinical evaluation; (4) incomplete clinical data or loss to follow-up; (5) benign paroxysmal positional vertigo (BPPV), persistent postural-perceptual dizziness (PPPD), nonspecific dizziness, or mixed vestibular disorders in which a definite primary diagnosis could not be established; and (6) central vertigo secondary to structural or other central nervous system disorders (e.g., stroke, multiple sclerosis, brain tumors, or other intracranial space-occupying lesions), as determined by clinical evaluation and neuroimaging when indicated.

### Method details

#### Treatment regimens

Patients received 75 mg rimegepant orally disintegrating tablets (Pfizer Investment Co., Ltd.; NMPA Approval No. HJ20240004) according to one of two dosing regimens: qd or eod. The dosing regimen was selected by the treating physician based on individual clinical characteristics and routine clinical practice. Alternate-day administration reflected the approved preventive dosing schedule, whereas daily administration represented an exploratory off-label dosing strategy used in selected patients. The safety of both regimens was informed by available clinical evidence and routine clinical experience.^48^^,^^49^ During the study period, the use of no more than one concomitant medication with potential migraine-prophylactic activity (e.g., topiramate or betahistine) was permitted, provided that the dose had remained stable for at least 3 months before inclusion and was not modified during follow-up.

#### Data collection

Demographic and clinical characteristics were systematically collected from medical records at baseline, encompassing primary symptoms (vertigo, headache, HL, and tinnitus) and associated clinical features. The severity of core symptoms was assessed using the NRS at baseline, week 4, and week 8. For the evaluation of psychological function, the SAS, SDS, and SCL-90 were administered solely at baseline and week 8. Additionally, hearing function was assessed at baseline and post-treatment during the 8-week follow-up period using pure-tone audiometry (air- and bone-conduction thresholds). PTA was calculated as the average air-conduction hearing threshold at 0.5, 1, 2, and 4 kHz. For patients with unilateral HL, the PTA of the affected ear was used, whereas for patients with bilateral HL, the mean PTA of both affected ears was calculated.

As an exploratory descriptive outcome, patients were asked to provide a global self-assessment of symptom improvement using four categories (marked, moderate, mild, or none). These patient-reported global assessments were collected at weeks 4 and 8 after treatment initiation and were not used as a validated primary efficacy outcome. The time to perceived onset of symptom improvement was also recorded based on patient self-report at the week 2 follow-up. The primary analyses were based on quantitative outcome measures rather than these patient-reported global improvement categories.

#### Outcomes

The primary outcome for the overall vertigo group was the number of monthly vertigo days.^50^ Secondary outcomes included psychological assessments (SAS, SDS, and SCL-90), as well as patient-reported time to perceived symptom improvement. For symptom-specific subgroups, the primary outcome was defined according to the predominant manifestation: PTA thresholds in patients with concomitant HL, tinnitus severity assessed by the NRS in those with tinnitus, patient-reported presence or absence of ear fullness in those with aural fullness, and the number of monthly migraine days in patients with comorbid migraine.

### Quantification and statistical analysis

All statistical analyses were performed using R software (version 4.4.0). Normality of continuous outcomes was assessed using the Shapiro-Wilk test. Normally distributed variables were expressed as mean (SD) and analyzed with two-sided paired t-tests; non-normally distributed variables were expressed as median with IQR and analyzed with two-sided paired Wilcoxon signed-rank tests. Hodges-Lehmann median differences and their 95% CIs were computed as robust effect size estimates for paired comparisons. Categorical outcomes were summarized as counts and percentages; specifically, changes in aural fullness from baseline to weeks 4 and 8 were assessed using two-sided exact McNemar tests.

To assess hearing improvement, a two-sided paired Wilcoxon signed-rank test was used to compare pre- and post-treatment PTA, with paired scatterplots and boxplots generated via ggplot2. For the association between rimegepant dosing and clinical outcomes, linear mixed-effects models (lme4, lmer) were fitted for monthly vertigo/headache days and tinnitus NRS scores, with dosing regimen as a fixed effect, patient as a random intercept, and adjustment for age, sex, BMI and follow-up duration. For the exploratory PTA dosing-regimen analysis, a generalized linear model was fitted with adjustments for age, sex, and BMI. For the binary aural-fullness outcome, logistic regression was used to examine the dosing-regimen association, with adjustment for age, sex, BMI, and follow-up duration.

Unless otherwise specified, n denotes the number of patients. The overall longitudinal analysis included *n* = 54 patients; paired audiometric analysis included *n* = 17; tinnitus, aural fullness, and migraine subgroup analyses included *n* = 37, *n* = 32, and *n* = 33, respectively; and the qd and eod groups included *n* = 28 and *n* = 26. Continuous data are reported as mean (SD) or median (IQR), as specified in the tables. Exact *n* values, tests, effect estimates, 95% CIs, and *p* values are reported in the results, tables, and figure legends. For the primary outcomes, effect size estimates together with their corresponding 95% CIs were additionally reported to provide quantitative measures of the magnitude and precision of observed associations. A two-sided *p* value <0.05 was considered statistically significant.
